# ZMPSTE24 Is Associated with Elevated Inflammation and Progerin mRNA

**DOI:** 10.3390/cells9091981

**Published:** 2020-08-28

**Authors:** Moritz Messner, Santhosh Kumar Ghadge, Thomas Maurer, Michael Graber, Simon Staggl, Sarah Christine Maier, Gerhard Pölzl, Marc-Michael Zaruba

**Affiliations:** 1 Department of Internal Medicine III, Cardiology and Angiology, Medical University Innsbruck, 6020 Innsbruck, Austria; moritz.messner@i-med.ac.at (M.M.); santhosh.ghadge@i-med.ac.at (S.K.G.); thomas.maurer@i-med.ac.at (T.M.); simon.staggl@i-med.ac.at (S.S.); gerhard.poelzl@i-med.ac.at (G.P.); 2 Department of Thoracic & Cardiac Surgery, Medical University Innsbruck, 6020 Innsbruck, Austria; michael.graber@i-med.ac.at; 3 Department of Medical Statistics, Informatics and Health Economics, Medical University Innsbruck, 6020 Innsbruck, Austria; sarah.maier@i-med.ac.at

**Keywords:** ZMPSTE24, lamin A/C, progerin, aging, inflammation

## Abstract

Lamins are important filaments forming the inner nuclear membrane. Lamin A is processed by zinc metalloproteinase (ZMPSTE24). Failure to cleave a truncated form of prelamin A—also called progerin—causes Hutchinson–Gilford progeria syndrome a well-known premature aging disease. Minor levels of progerin are readily expressed in the blood of healthy individuals due to alternative splicing. Previously, we found an association of increased progerin mRNA with overweight and chronic inflammation (hs-CRP). Here, we aimed to elucidate correlations of ZMPSTE24, lamin A/C and progerin with the inflammatory marker hs-CRP. In this retrospective, cross-sectional study we analyzed blood samples from 110 heart failure patients for quantitative mRNA expression of ZMPSTE24, lamin A/C, progerin and hs-CRP protein. Spearman correlations and linear regression analyses including adjustments for age, gender and ejection fraction showed a significant positive correlation of lnprogerin with lnZMPSTE24 (n = 110; r = 0.33; *p* = 0.0004) and lnlamin A/C (n = 110; r = 0.82, *p* < 0.0001), whereas no association was observed between lnlamin A/C and lnZMPSTE24 expression. Further analyses showed a significant positive correlation of lnhs-CRP with lnZMPSTE24 (n = 110; r = 0.21; *p* = 0.01) and lnlamin A/C (n = 110; r = 0.24; *p* = 0.03). We conclude that chronic inflammation is associated with increased expression of ZMPSTE24 and lamin A/C mRNA. Both markers also positively correlate with increased expression of the premature aging marker progerin which may be linked to cardiovascular aging.

## 1. Introduction

Nuclear lamins are structural proteins and as type V intermediate filaments involved in major functions like cell-cycle regulation, DNA-replication, signal transduction and gene expression, as well as chromatin and pore positioning [1].

Defects while processing lamin A could lead to the premature aging syndrome Hutchinson–Gilford progeria (HGPS) [2]. Individuals suffering from HGPS die as a result of severe atherosclerosis, either fatal stroke, myocardial infarction or heart failure generally before the age of 20 [3].

**Figure 1:**
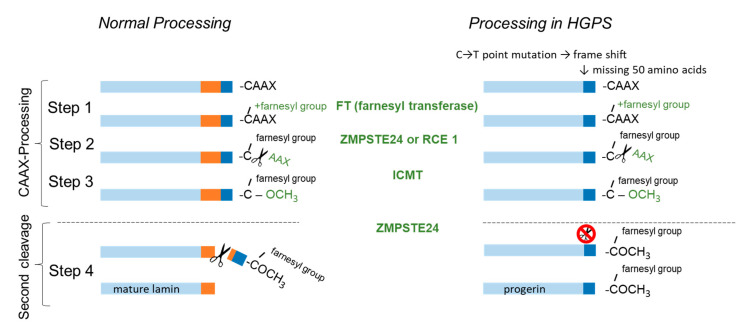
Lamin A biogenesis consists of four steps of post-translational processing. In Step 1 to 3 the C-terminal CAAX motiv is processed by farnesyl transferase (FT), ras-converting enzyme (RCE1) or zinc-metalloproteinase 24 (ZMPSTE 24) and isoprenylcysteine carboxyl methyl transferase (ICMT). In the final step the farnesyl anchor is cleaved by ZMPSTE24 to form mature lamin A protein. The point-mutation LMNA 1824 C > T, G608G in exon 11 activates a cryptic splicing site which results in a truncated prelamin A protein (Δ50 aa) also called progerin lacking the cleavage site for ZMPSTE24.

To process mature lamin A from its precursor, prelamin A, four post-translational modifications are necessary (Figure 1): In the first step, the C-terminal CAAX motif (C = cysteine sulfhydryl, A = aliphatic amino acid, X = amino acid other than proline) is farnesylated [4]. Following the farnesylation of cysteine sulfhydryl, AAX is proteolytically cleaved by the Ras-converting enzyme (RCE1) or zinc–metalloproteinase 24 (ZMPSTE 24) (Step 2), and in Step 3 the remaining farnesylated cysteine undergoes carboxyl methylation through isoprenylcysteine carboxyl methyl transferase (ICMT). After the process of farnesylation, lamins are targeted to the nucleus, where they interact with the inner nuclear membrane. A final cleavage (Step 4) by ZMPSTE 24 is fundamental to form functional lamin A [5,6].

In patients with HGPS, ZMPSTE24 is unable to cleave the farnesylated and carboxyl methylated residue leading to accumulation of the truncated premature protein progerin in the nuclear periphery disabling its fixation into the nuclear lamina. As a consequence nuclei are morphologically misshaped and its proper functioning is severely impaired [7]. The cause for HGPS is most prevalently a point-mutation in the lamin A gene leading to a frameshift which results in a protein lacking the cleavage site for ZMPSTE24 [8]. Mutations causing an enzyme dysfunction in ZMPSTE24 also result in HGPS related premature aging disorders like mandibuloacral–dysplasia type B and restrictive dermopathy.

Expression of low levels of progerin through alternative splicing was found in non HGPS cells and was previously linked by Scaffidi et al. with physiologic aging [7]. Consecutively, progerin is discussed by various groups as a marker for cardiovascular aging. Since laminopathies are a known cause of cardiomyopathies, we recently found a positive correlation between progerin mRNA expression and LV dysfunction in patients with dilated cardiomyopathy [9]. Furthermore, we previously found an association of obesity and inflammation (hs-CRP) with high levels of progerin mRNA expression [10].

Therefore, we aimed to elucidate correlations of ZMPSTE24, lamin A/C and progerin mRNA expression with the inflammatory marker CRP.

## 2. Materials and Methods

### 2.1. Study Population

We performed a cross-sectional retrospective analysis enrolling patients presenting at our cardiology outpatient clinic between 2014 and 2016. We consecutively included 110 Caucasian patients over 18 years of age of both sexes. Diagnoses of heart failure etiology are depicted in Table A1. Most of the patients suffered from DCM, followed by ICM, hypertrophic cardiomyopathy and other reasons for heart failure [10].

For measurement of ZMPSTE24, lamin A/C and progerin mRNA levels, EDTA blood samples were obtained from 110 patients.

All included individuals with chronic heart failure were treated according to the current guidelines [11]. Moderate to severe chronic kidney disease, acute heart failure and imminent heart transplantation were exclusion criteria.

The study complied with the principles set out in the Declaration of Helsinki and was approved by the local ethics committee of Innsbruck Medical University with the approval code AM4077 (approved on 24 August 2010). All patients gave written informed consent for participation in the analyses.

### 2.2. RT–PCR of mRNA

Blood samples were collected into anticoagulant (EDTA) tubes and centrifuged for 10 min at 3600× *g*. After centrifugation, the buffy coat enriched with leukocyte fraction was extracted, and lysed in red blood lysis solution. Cells were washed, resuspended in Trizol (Invitrogen) and stored at −80 °C until analysis. Total mRNA was extracted, and reverse transcribed into cDNA using QuantiTect RT kit (Qiagen GmbH, 40,724 Hilden, Germany) as per the manufacturer’s protocol.

**Figure 2:**
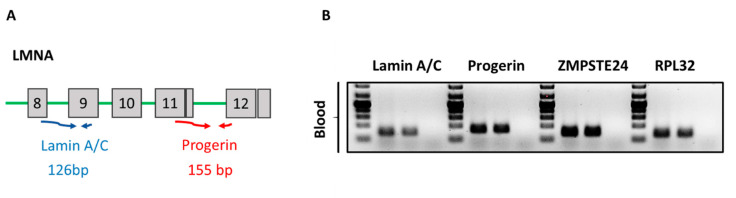
(**A**) Primers spanning exon 8 to 9 of the LMNA gene were created to detect lamin A/C mRNA levels as depicted in diagram A. To specifically detect progerin primers spanning the cryptic splice site and exon 11 and 12 were designed; (**B**) agarose gels showing ZMPSTE24, lamin A/C and progerin PCR products from two human blood samples. RPL32 was used as a housekeeping gene.

For mRNA quantification of progerin and lamin A/C gene, specific primers were used which were published previously by us (9). ZMPSTE24 gene specific primers were designed and optimized. Rpl32 was used as a housekeeping gene. The amplified PCR products were verified by sequencing and agarose gel analysis (Figure 2B). The sequences of PCR primers were:

Rpl32-F: 5′-AGTTCCTGGTCCACAACGTC-3′,

Rpl32-R: 5′-CTCTTTCCACGATGGCTTTG-3′,

RPL32 F: 5′-AGTTCCTGGTCCACAACGTC-3′,

Progerin F: 5′-TCAGGAGCCCAGAGCCCCCAGAAC-3′,

Progerin R: 5′-GGGTTATTTTTCTTTGGCTTCA-3′,

LaminA/C F: 5′-GGTGGTGACGATCTGGGCT-3′,

LaminA/C R: 5′-CCAGTGGAGTTGATGAGAGC-3′.

ZMPSTE24-8F: 5′-TCGCTGTACTAGGCCATGAA-3′

ZMPSTE24-9R: 5′-CAAAAAGCTCCTTTCGACCA-3′

The cycling conditions for qPCR were 95 °C for 10 min (Activation), 95 °C for 15 sec (denaturation) and 60 °C for 1 min (Annealing and extension) up to 40 cycles. For PCR, 2× SYBR green master mix (Applied Biosystems, Waltham, MA 02451, USA) was used, and the average Ct (cycle threshold) was calculated out of two sample of the same probe. Expression levels were normalized to the reference gene RPL32 and quantitative gene expression was calculated with comparative ΔΔCt method.

### 2.3. Statistical Analysis

Statistical analyses were conducted with SSPS™ Statistics 24.0.0 (IBM, Armonk, NY, USA) and GraphPad Prism (version 6.04, GraphPad Software, Inc., San Diego, CA 92108, USA) was used to generate graphics. Quantitative variables of the baseline characteristics are expressed as means ± standard deviation (SD) and categorical variables are shown as absolute values with percentages in brackets. Due to a right skewed distribution of CRP, ZMPSTE24 mRNA, lamin A/C mRNA and progerin mRNA, the natural logarithm (ln) of the variables was used to achieve approximative normality by ln-transformation. Pearson’s (for normal distributed variables) and Spearman correlation coefficients were calculated, and scatter plots were created. In addition, to consider possible confounding by key demographic and clinical characteristics, linear regression analyses adjusting for age, gender and ejection fraction were performed.

## 3. Results

### 3.1. Detection of ZMPSTE24, Lamin A/C and Progerin mRNA in Human Blood Samples

Figure 2A depicts the schematic PCR based strategy to analyze expression of lamin A/C and progerin mRNA expression in human blood samples. Primers spanning exons 8 to 9 of the LMNA gene were used to measure total lamin A/C mRNA levels. To specifically detect progerin mRNA, primers spanning the cryptic splice site between the exons 11 and 12 were designed. As shown in Figure 2B, lamin A/C, progerin, and ZMPSTE24 PCR products were readily detectable in human blood samples.

Further sequencing of the purified specific progerin PCR product revealed the anticipated gene sequence confirming alternative splicing of progerin in the blood.

### 3.2. Characteristics of Study Cohort

Detailed patient characteristics on admission are shown in Table 1. During the study period, blood samples from 110 patients were collected. The mean age of the study participants was 56 ± 15 years; 32 patients (29%) were female. The mean eGFR was 54 ± 10. Diagnoses of heart failure etiology are depicted in Table A1. Most of the patients suffered from DCM (53%), followed by ICM (28%), hypertrophic cardiomyopathy (11%) and other reasons for heart failure (8%).

### 3.3. Lamin A/C and ZMPSTE24 mRNA Correlate with Progerin mRNA

**Figure 3:**
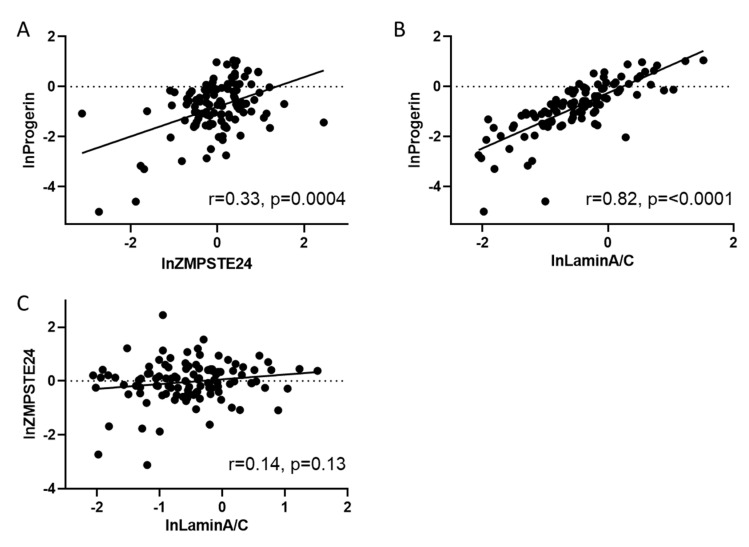
Progerin correlates to ZMPSTE24 and lamin A/C. (**A**) Scatter plot showing the positive correlation (r = 0.33) between lnprogerin mRNA related to lnZMPSTE24 mRNA in human blood samples (n = 110) *p* = 0.0004; (**B**) scatter plot showing the positive correlation (r = 0.82, *p* = < 0.0001) between the relative amount of lnlamin A/C mRNA related to RPL32 with lnprogerin in human blood samples (n = 110); (**C**) no significant correlation was observed between lnlamin A/C and lnZMPSTE expression.

Since ZMPSTE24 is involved in the critical cleavage step of prelamin A to lamin A, we were interested whether there was an association of ZMPSTE24, lamin A/C and the premature aging-related splice variant progerin. LnZMPSTE24 mRNA (n = 110; r = 0.33; *p* = 0.0004) (Figure 3A) as well as lnlamin A/C (n = 110; r = 0.82, *p* < 0.0001) mRNA expression (Figure 3B) was significantly positively correlated with lnprogerin mRNA expression levels. Correlations remained highly significant even after adjusting for age, gender and ejection fraction using linear regression analysis (standardized coefficient = 0.461, *p* < 0.001 for ZMPSTE24mRNA vs. progerin mRNA, and stand. coeff. = 0.747, *p* < 0.001 for laminA/C mRNA vs. progerin mRNA). No association was observed between lamin A/C and ZMPSTE mRNA expression (Figure 3C).

### 3.4. CRP Correlates with Expression of Lamin A/C and ZMPSTE24 mRNA

**Figure 4:**
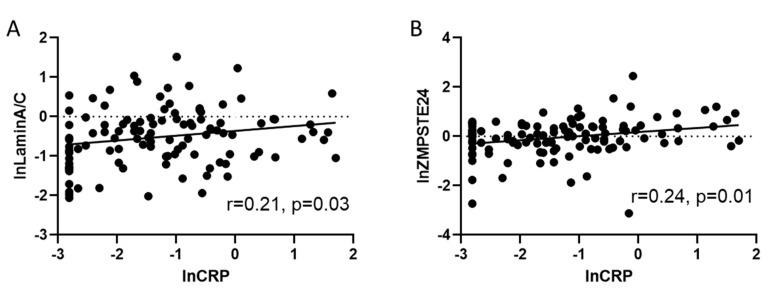
CRP positively correlates to lamin A/C and ZMPSTE. (**A**) Scatter plot showing a weak positive correlation (r = 0.21) between lnCRP related to lnlamin A/C in human blood samples (n = 110) *p* = 0.03; (**B**) scatter plot showing the positive correlation (r = 0.24) between the relative amount of lnZMPSTE24 mRNA related to RPL32 with lnCRP in human blood samples (n = 110). Both correlations remained significant after adjusting for age, gender and ejection fraction with linear regression analysis.

Next, Spearman’s correlations (n = 110) were performed to calculate whether inflammation reflected by hs-CRP serum levels were correlated to total lamin A/C or ZMPSTE mRNA expression. As shown in Figure 4A, lnlamin A/C mRNA expression was positively related to lnserum–CRP levels (n = 110; r = 0.24; *p* = 0.03). Although the correlation between lnlamin A/C mRNA and lnCRP was weak, adjustment for age, gender and ejection fraction using linear regression analysis still revealed a statistically significant correlation (stand. coefficient = 0.215, *p* = 0.032 for lnlaminA/C vs. lnCRP) suggesting at least a weak association.

Furthermore, a significantly positive correlation was observed between lnZMPSTE and lnhs-CRP (n = 110; r = 0.21; *p* = 0.01) (Figure 4B). The correlation remained highly significant even after adjusting for age, gender and ejection fraction using linear regression analysis (stand. coeff. = 0.282, *p* = 0.003 for lnZMPSTE24 vs. lnCRP).

## 4. Discussion

**Figure 5:**
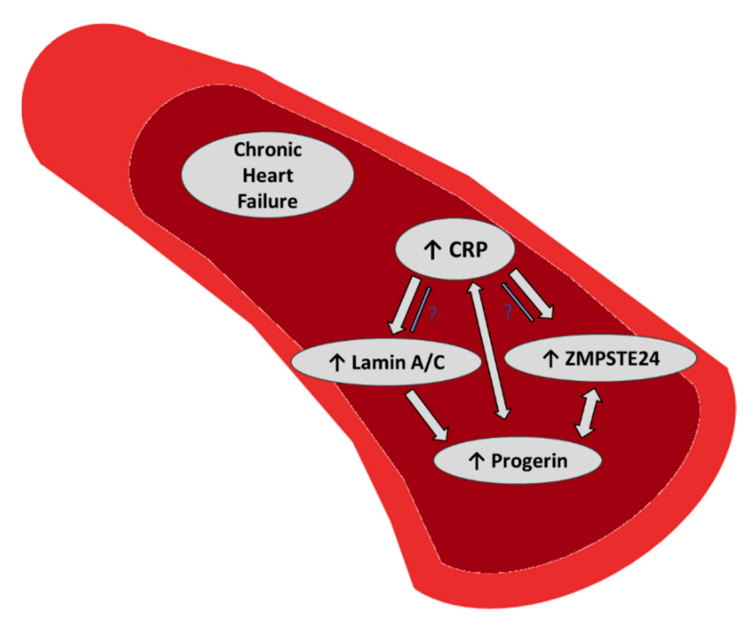
Summary of our previous and recent findings: In a heart failure outpatient cohort we found a positive correlation of the premature aging marker progerin with lamin A/C and ZMPSTE24 mRNA, which may link both markers also with cardiovascular aging. Moreover, chronic inflammation (CRP) was positively associated with increased expression levels of progerin [10], lamin A/C and ZMPSTE24 mRNA, providing a possible link of premature aging with inflammation.

Our study in a heart failure outpatient cohort including 110 patients shows that mRNA levels of the lamin A processing zinc metalloproteinase ZMPSTE24 and lamin A/C were significantly positively correlated to expression of the premature aging-related lamin A splice variant progerin mRNA, suggesting that both markers may also be linked to cardiovascular aging. Moreover, ZMPSTE24 and lamin A/C were both positively correlated to hs-CRP (Figure 5).

Since we previously also found a positive association between progerin mRNA, overweight (body mass index), and inflammation (hs-CRP) our data may suggest a positive link between cardiovascular aging and inflammation in patients with heart failure [10]. Increased progerin mRNA expression was related to LV dysfunction, and progerin expression in the nucleus of cardiomyocytes was related to apoptotic cell death in patients with dilative cardiomyopathy [9].

A link between obesity, aging and chronic inflammation was also reported previously by other groups [12,13,14]. A recently published study showed that two missense heterozygous mutations in LMNA, c.1745G > T Arg582Leu and c.1892G > A Gly631Asp, were associated with severe metabolic phenotypes and nuclear misshaping [15].

ZMPSTE24 catalyzes the enzymatic cleavage of prelamin A to functional mature lamin A. This process is disturbed in HPGS due to a point mutation in the lamin A gene leading to a frameshift which results in a defective protein called progerin lacking the cleavage-site for ZMPSTE24. The enzyme ZMPSTE24 is known to be involved in critical steps of lamin A processing and its proper functioning is crucial for the production of a mature protein. Mutations of ZMPSTE24 were found to reduce this processing and cause severe progeroid disorders similar to mutations of the LMNA gene leading to HGPS [16]. From a mechanistic point of view, linking progerin to ZMPSTE24 mRNA expression seems of special interest. Therefore, we hypothesized that expression levels of ZMPSTE24, lamin A/C and progerin were somehow correlated to each other. Indeed, in our study cohort, progerin mRNA expression was positively correlated with ZMPSTE24 mRNA suggesting ZMPSTE24 as a possible marker to detect premature aging in the cardiovascular system.

The reason for the positive correlation of progerin with ZMPSTE24 is not fully clear. However, one explanation may be an upregulation of ZMPSTE24 to compensate for increased amounts of cleavage resistant progerin. In line with this assumption, we could not find a significant correlation of lamin A/C mRNA with ZMPSTE24 expression, although there was a slight trend and a priori it would be assumed, that increased lamin A/C levels may influence ZMPSTE24 expression levels. However, based on our data no conclusion on the causality can be drawn.

In our cohort, we also found an association between the inflammation biomarker hs-CRP and the expression of ZMPSTE24 mRNA. In this regard, a recently published study links inhibition of ZMPSTE24 activity by protease treatment in HIV patients with increased levels of premature prelamin A mediating myocardial inflammation and HIV-associated cardiomyopathy [17].

Liu C et al. demonstrated that 17 genes involved in atherosclerosis, arthritis, lipodystrophy and hair loss were altered in HGPS, in normal cells during senescence and during DNA damage induced senescence. Fourteen out of this genes are known to encode for proinflammatory markers [18]. Furthermore, Osorio et al. found that accumulation of prelamin A isoforms at the nuclear lamina leads to NF-κB activation and secretion of high levels of proinflammatory cytokines in two different mouse models of accelerated aging (Zmpste24(-/-) and Lmna(G609G/G609G) mice) [19].

We also found a positive correlation of lamin A/C with progerin mRNA and hs-CRP. It seems plausible that an increase in common gene transcription also leads to higher amounts of its alternatively spliced products. Fitting this hypothesis, we found that higher levels of lamin A/C were correlated with increased amounts of progerin mRNA. An upregulation of lamin in obese was already described by Miranda et al. in a small cohort [20]. In human adipose tissue expression of lamin A/C mRNA was positively related with BMI and inflammatory IL6 gene expression. In the same study, overexpression of lamin A/C in mouse macrophages revealed chronic inflammation by upregulation of mediators like IL6, TNFα and CCL2 and the development of obesity-induced insulin resistance [21].

Based on these observations, the hypothesis could be postulated that abundant lamin A/C expression, e.g., due to specific conditions like chronic inflammation or obesity affects the whole body or single organs like the heart by contributing to aging related processes.

### Limitations

The investigated population was limited to patients of a cardiology outpatient unit implying impaired generalizability concerning other cohorts and normal controls. However, our cohort consisted of cardiovascular diseases associated with premature aging and limited longevity.

The methods used in this study do not provide sufficient information about protein levels or genetics and epigenetic varieties in the individuals and are limited to mRNA measurements by RT–PCR. Further studies in larger patient cohorts are warranted to elucidate the exact roles and interactions of ZMPSTE24, lamin A/C and progerin in the process of cardiovascular aging. Furthermore, the use of primers specific for different types of lamin may provide additional insights.

## 5. Conclusions

We conclude that chronic inflammation is associated with increased expression of lamin A/C and ZMPSTE24 mRNA. Both markers were also positively related with the expression of the premature aging marker progerin and thus may be linked to accelerated cardiovascular aging.

## Figures and Tables

**Table 1:** Baseline characteristics of the study cohort.

Characteristic	Total Population N = 110	
Age, years		56 ± 15
Female sex, n (%)		32 (29)
Body mass index, kg/m^2^		26.6 ± 4.8
NYHA class, n (%)	N = 108	2.0 ± 0.7
I		29 (26)
II		55 (50)
III		24 (22)
IV		0 (0)
Medical history, n (%)		
Smoking, n (%)	N = 108	49
Diabetes, n (%)		19 (17)
Atrial fibrillation, n (%)		42 (38)
LVEF, n (%)		38.2 ± 14.9
ACE-inhibitor, n (%)		55 (50)
ARB, n (%)		33 (30)
Beta blocker		90 (81)
Clinical features		
NT-proBNP, ng/L	N = 108	2094 ± 4421
Troponin T, ng/L	N = 109	28 ± 53
Serum creatinine, mg/dl		1.2 ± 0.46
GFR, mL/min		54 ± 10
Urea, mg/dl		50 ± 37
HbA1c,%	N = 110	5.9 ± 1.0
Triglycerides, mg/dl		135 ± 68
Total cholesterol, mg/dl		172 ± 44
LDL, mg/dl	N = 109	107 ± 37
HDL, mg/dl	N = 109	52 ± 19
Hs-CRP, mg/dl	N = 110	0.64 ± 1.07
GGT, U/I		86 ± 146
GOT, U/I		29.2 ± 12.4
Potassium		4.2 ± 0.5
Sodium		138 ± 13
Leucocyte count, G/L		7.7 ± 2.2
Thrombocytes, G/I		217 ± 71
Hemoglobin, g/L		140 ± 15

Plus–minus values are means ± standard deviation. NYHA—New York Heart Association class; LVEF—left ventricular ejection fraction; ARB—angiotensin receptor blocker; GFR—glomerular filtration rate; LDL—low-density lipoprotein; HDL—high-density lipoprotein; Hs-CRP—high-sensitivity C-reactive protein; GGT—gamma-glutamyltransferase; GOT—glutamic oxaloacetic transaminase.

## Abbreviations

BMI	Body mass index
CVS	Cardiovascular system
DCMP	Dilative cardiomyopathy
HGPS	Hutchinson–-Gilford progeria syndrome
Hs-CRP	High-sensitive C-reactive protein
HCMP	Hypertrophic cardiomyopathy
ICMP	Ischemic cardiomyopathy
NYHA	New York Heart Association
PCR	Polymerase chain reaction

## Appendix A

**Table A1:** Diagnosis of included patients.

DCMP	ICMP	HCMP	Others	Total
58 (53)	31 (28)	12 (11)	9 (8)	110

DCMP—dilative cardiomyopathy; ICMP—ischemic cardiomyopathy; HCMP—hypertrophic cardiomyopathy.
